# Antibody-mediated phosphatidylserine blockade significantly enhances the efficacy of downstream immune checkpoint inhibition in K1735 mouse melanoma

**DOI:** 10.1186/2051-1426-2-S3-P205

**Published:** 2014-11-06

**Authors:** Xianming Huang, Jian Gong, Dan Ye, Van Nguyen, Shen Yin, Rich Archer, Chris Hughes, Rolf Brekken, Jeff Hutchins, Alan Schroit, Bruce Freimark

**Affiliations:** 1 Department of Pharmacology, UT Southwestern Medical Center, Dallas, TX, USA; 2 Peregrine Pharmaceuticals, Inc, Tustin, CA, USA; 3 Department of Surgery, University of Texas, Southwestern Medical Center at Dallas, TX, USA; 4 Peregrine Pharmaceuticals, Inc, USA; 5 University of California, Irvine, CA, USA; 6 Department of Clinical Affairs, Peregrine Pharmaceuticals Inc., Tustin, CA, USA

## 

Phosphatidylserine (PS) is an upstream immune checkpoint that drives global immunosuppression. Previous work has shown that PS targeting agents can override PS-driven immunosuppression and re-program the tumor microenvironment from immunosuppressive to immunosupportive, break tumor immune tolerance, and elicit potent *de novo *antitumor T-cell immunity. In the present study, the antitumor effect of the combination of a PS-targeting antibody with antibodies that inhibit the downstream immune checkpoints PD-1 or CTLA-4 antibody in the K1735 mouse melanoma model was examined. Tumor-bearing mice were treated with each antibody alone or the combination at 5 to 10 mg/kg, twice a week. Combination therapy potently suppressed tumor growth and improved overall survival compared to single agent treatment. Flow cytometry revealed that combination therapy induced the highest ratio of tumor-infiltrating immune effector to suppressor cells. Importantly, combination treatment also significantly decreased the levels of myeloid-derived suppressor cells (MDSC) in the spleen. In addition, inhibition of PS and PD-1 or CTLA-4 resulted in significantly more IL-2 and IFNg-secreting splenic CD4+ and CD8+ T cells than any single agent treatment. Finally, combined immune checkpoint blockade did not induce any observable toxicity following multiple treatment doses. In summary, our findings demonstrate that the combination of antibody-mediated PS blockade with an inhibition of established immune checkpoints (e.g., PD-1 and CTLA-4) represents a promising strategy for cancer immunotherapy.

